# Analysis of Nutritional Supplements Consumption by Squash Players

**DOI:** 10.3390/nu10101341

**Published:** 2018-09-20

**Authors:** Anna Ventura Comes, Antonio J. Sánchez-Oliver, José Miguel Martínez-Sanz, Raúl Domínguez

**Affiliations:** 1 Faculty of Health Sciences of Universidad Isabel I, Universidad Isabel I, 09004 Burgos, Spain; anna.ventura@alumnos.ui1.es (A.V.C.); raul.dominguez@ui1.es (R.D.); 2 Faculty of Sports Sciences of Universidad Pablo de Olavide, Universidad Pablo de Olavide, 41004 Sevilla, Spain; 3 Human Motricity and Sports Performance Area, University of Seville, 41004 Sevilla, Spain; 4 Nursing Department, Faculty of Health Sciences, University of Alicante, 03080 Alicante, Spain; josemiguel.ms@ua.es

**Keywords:** sport nutrition, nutritional supplements, performance, squash, ergogenics aids

## Abstract

The aim of the present study is to analyse nutritional supplements (NS) consumption by squash players of different levels (international vs. national). A total of 14 international players and 28 national ones answered a NS consumption frequency questionnaire that had been previously validated. A T-Student test was used for independent samples and a χ^2^ test was used in the comparative analysis among athletes of different levels. International level players consume NS in greater proportion vs. national level players (100% vs. 67.9% *p* = 0.017), with differences in the consumption of bicarbonate of soda, glutamine, branched-chain amino acids, and flaxseed oil (*p* < 0.05). Even though international level athletes show a greater total number of NS, as well as of the total of NS of each of the categories based on scientific evidence level (sports food, medical supplements, and ergogenic aids of groups A, B, C, and D) in relation to the national level athletes, no statistically significant differences were detected (*p* > 0.05). With regard to nutritional advice, there are also differences among performance levels (*p* = 0.003), being personal trainers (28.6%) and dietitians-nutritionists (21.4%) the greatest prescribers when it comes to international-level players, whereas 55.6% of the national-level players do not receive nutritional advice. The pattern of NS consumption, based on evidence level, is unbalanced and its performance could be favored if the dietitian-nutritionist were included as a nutritional advisor for these athletes.

## 1. Introduction

Squash, with its origins in the courtyards of London Fleets prison in the 18th century, is one of the four big racquet sports, together with tennis, badminton, and table tennis, and it is practiced by ~20 million people worldwide. Squash is a sport that requires multiple sprints and hitting during which metabolism is asked for phosphocreatine [1]. Nevertheless, the shortest recovering periods between high intensity actions together with the longest duration of such efforts, as compared to other racquet sports, cause a progressive depletion of phosphocreatine reserves during a game [1], as well as a rise of the glycolytic pathway that may lead to a pH decrease [2], and both factors can therefore act as performance limiting factors. Moreover, squash players need a high aerobic strength, since the average oxygen consumption during a game is 86–92% of the maximum oxygen consumption (VO_2_max) [3]. These metabolic demands translate to high resistance requirements, both aerobic and anaerobic, muscular strength and manual grip strength, movement speed, change of direction and reaction, agility, acceleration, and flexibility [2,3,4]. Psychological variables, such as mental strength and decision making on the shot and the play, must be added to these metabolic demands [3,4].

When the performance level increases considerably, adequate intake of energy and nutrients becomes even more critical as any small benefit acquired can provide an advantage during the competition. Apart from high metabolic and physical requirements of sport, we should also consider that the equality of competition is growing and that improvements of just 0.6% can have a direct impact on the results of a competition [5]. This possibility of improving performance often encourages athletes to consider nutritional supplements (NS) consumption. NS destined for athletes are becoming increasingly important and their consumption has been growing exponentially in recent years [6]. There are many NS on the market and their numbers also continue to grow vertiginously. Some NS are presented as solid foods, others as drinks, and still others in concentrated and dosed forms. Although NS use is widespread in the world of sports, only a few NS have been shown to result in improved sports performance [7].

Therefore, in 2000, the Australian Institute of Sport (AIS) created a NS classification system for athletes (known as ABCD system), in which NS are differentiated according to the existent level of scientific evidence, as well as to other parameters that are related to security, legality, and effectiveness to improve sports performance [7]. Thus, NS belonging to group A constitute NS that show a high level of scientific evidence for performance improvement, distinguishing among sports food (it provides a source of nutrients when it is not practical to consume everyday food, such as sports drinks, bars and gels, or milk whey protein), medical supplements (effective when treating clinical alterations such as nutritional deficiency) and performance supplements (they improve sports performance: caffeine, B-alanine, creatine, sodium bicarbonate, and nitrate or beetroot juice) [7]. On the other hand, group B comprises NS that might have a positive effect on certain conditions, although a higher number of investigations is required, whereas group C comprises NS with no ergogenic effects and group D comprises NS of which use is not allowed due to being included in the list of substances and methods forbidden in sports. In any case, even amongst NS with a high level of scientific evidence on performance improvement, such as caffeine, creatine, β-alanine, sodium bicarbonate, and beetroot juice/nitrate [8], ergogenic effects are subject to dosage, as well as to metabolic and mechanical demands of effort [9].

Besides the possibility of improving sports performance, the intake of NS may carry certain risks as there is a lack of such information in the literature [10] and current Spanish legislation regarding NS is both non-specific and limited [11]. In addition, as NS are often purchased via the internet with issues that are related to their safety and legality [12], a percentage of NS could contain banned or harmful substances [11]. Moreover, the interest of some in consuming NS to fulfill proposed targets despite harmful effects on their health complicates matters even further [13].

Maughan [8] proposes that any dietetic and nutritional intervention must be done based on dietetic measures that consider the nutritional goals of the sports category, which, in turn, are established based on the limiting factors of such sports category. Therefore, after analyzing the metabolic demands and the factors that limit performance in squash and given that there are no prior researches that have analyzed NS prevalence and consumption by squash players at a competitive level, the aim of the current paper is to analyse the pattern of NS consumption and choice by squash players who compete at a national and international level.

## 2. Materials and Methods

### 2.1. Participants

For recruiting the sample, Real Federación Española de Squash and the 14 autonomic federations sent an email informing the athletes about the execution of this study and inviting the athletes in the senior category who had competed in the national competition to collaborate. Eventually, a total of 42 players (29 men and 13 women) participated in the study. The sample includes 100% of the Spanish players who compete at an international level, either because they compete in the worldwide professional circuit with the Professional Squash Association permission or because they are members of Absolute Spanish Team of Squash. Therefore, the sample was divided into two groups: one group comprising international-level players (14 players, 10 of which are males and four are females) and another one composed of national-level players (28 players, 20 of which are males and eight are females). This research study was approved by the Ethics Comitte of Alfonso X El Sabio University.

### 2.2. Procedure

The athletes, who voluntarily agreed to participate in this study, were sent a telematic survey on NS consumption previously validated, in which the validity of its contents was assessed, noting the instrument’s capacity to measure that for which it was constructed; its application, analyzing benefits and shortcomings and revising the instrument’s instructions; its structure, revising the formulation of the questions, the sequence proposed and the response scale; and, its presentation, in which the best characteristics of the instrument’s appearance and format were identified [14]. The survey is organized in three parts: one in which the survey respondent’s anthropometric, personal and social data are gathered, a second one that aims to analyse the sports activity and its contextualisation, and a third part that is related to NS consumption and their possible repercussions on health. With regard to the use of NS, the questionnaire recorded the NS consumed in general and those currently ingested (current sports season), including questions about what NS they consumed, when they took them (before, during, and after training, and/or/competition?), and the time of consumption (training, competition or both). In addition information was collected about possible improvements noted, and subjects were asked to specify which NS had produced ergogenic effects and which ones had not. On a more personal level, they were asked about the consumption of banned substances and how they felt about the use of these substances in their sport. Also, this survey achieves a 54% of methodological quality in a review performed by Knapik et al. [6], in which only 57 of the 164 different questionnaires they reviewed for the study of NS consumption received their approval.

### 2.3. Statistical Analysis

Participants data and quantitative data, as well as the amount of NS consumed, are expressed as the value of the median (M) ± standard deviation (SD), whereas for the rest of variables, frequencies and percentages are used. In order to carry out a comparative analysis among quantitative variables of international-level players in relation to national-level ones, a T-Student test for independent samples was done, after having checked the normality using the Shapiro-Wilk test. On the contrary, in order to compare the differences in frequencies among players of different levels (international vs. national) a χ^2^ test was used. When a frequency under 5 is expected, it was applied the criterion *p* > (5/*n*), so all supplements showing a *p* < (5/*n*) were excluded. The level of statistical significance was set at *p* < 0.05. The statistical analysis was done while using the statistical package SPSS v.18.0 (SPSS Inc., Chicago, IL, USA).

## 3. Results

### 3.1. Descriptive and Analytical Data of Sports Practice

Table 1 shows descriptive and training frequency values of the sample, in which it is visible that international level-players are younger than those who play at a national level (25.0 years ± 6.2 vs. 35.6 ± 14.2, *p* = 0.01), while they carry out 43.2% more of training sessions per week (5.57 ± 1.16 vs. 3.9 ± 1.6, *p* < 0.01).

### 3.2. Data on Nutritional Supplements Consumption

**Figure 1:**
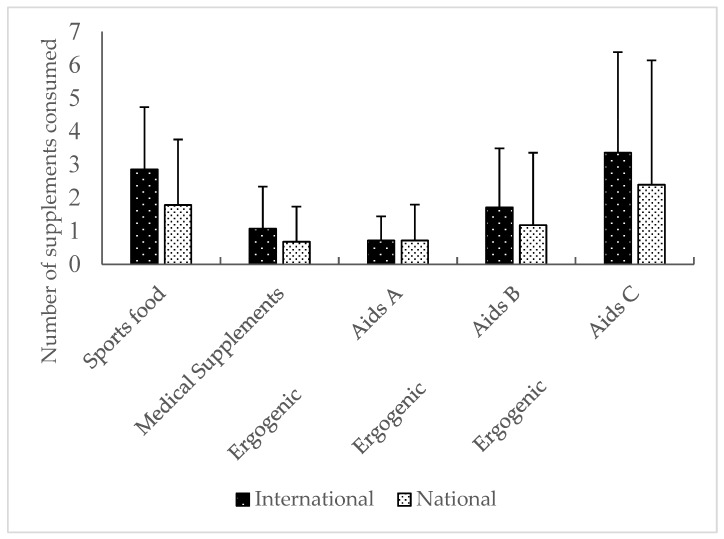
Number of supplements consumed by the international-level and national-level athletes’ group.

In relation to NS intake, 100% of the international-level players appeared to consume nutritional supplements, as opposed to 67.9% of the national-level players (19/28) (*p* = 0.02). Regarding the number of NS consumed by the athletes, even though the average of the supplements intake total by athlete is 54.5% higher in international-level players as compared to national-level players (10.2 + 8.0 vs. 6.6 + 9.2 supplements), no statistically significant differences were detected (*T* = 1.25, *p* = 0.51). With regard to the categorization of the different NS according to the AIS (AIS), despite the number of supplements that were consumed within the group of international-level players being higher in all categories (Figure 1), no statistically significant differences were detected in the supplements so-called sports food (*T* = 1.69, *p* = 0.10), in medical supplements (*T* = 1.06, *p* = 0.29) nor in ergogenic aids of groups A (*T* = 0.00, *p* = 1.0), B (*T* = 0.80, *p* = 0.43), and C (*T* = 0.84, *p* = 0.41).

Table 2 shows a comparative analysis of the supplement consumption of the different NS included in the different groups categorized by the AIS. This way, although the T-Student test for independent samples doesn’t show statistically significant differences in the total supplements consumption nor in the different groups of international and national-level athletes, the χ^2^ test showed differences in the consumption of bicarbonate of soda, flaxseed oil, glutamine, and branched-chain amino acids supplements. This way, international-level players have a higher intake of flaxseed oil (28.6% vs. 3.6%, *p* = 0.02), sodium bicarbonate (28.6% vs. 3.6%, *p* = 0.04), glutamine (50% vs. 17.9%, *p* = 0.03), and branched-chain amino acids (57.1% vs. 21.4%, *p* = 0.02) supplements in relation to national-level players.

Regarding advice on NS intake, statistically significant differences were found among groups (*p* < 0.01), being personal trainers (4/14) and dietitians-nutritionists (3/14) the greatest prescribers when it comes to the international-level players, whereas it is noteworthy that 55.6% of the national-level athletes who follow a diet declare that they do not receive advice (5/9) and the rest go to the dietitian-nutritionist (2/9) or read blogs (2/9).

## 4. Discussion

Our first research finding was that all the international-level players consume NS, so that the consume prevalence is higher than the one observed in international-level players of tennis, in which it reaches 81% [15]. In addition, it has been found that consumption among international-level players is significantly higher than among national-level players (*p* = 0.02) whose consumption rate is below 70%, which is confirmed by the results that were brought by a meta-analysis that concluded that elite athletes show a higher NS consumption rate [6].

In relation with sports food, it was found that the most consumed ones were energy bars and isotonic drinks, which were consumed by more than 70% of the international-level players and by half of the national-level players, followed by whey protein, which is consumed by 43% of the international-level players and by less than 30% of the national-level ones. Isotonic drinks promote hydration, by replacing electrolytes lost during the effort (sodium, potassium, calcium, and magnesium), and they delay the depletion of glycogen and hypoglycaemia, by providing a small quantity of carbohydrate (6–8 g/100 mL) [16]. In a sport such as squash, which represents a considerable challenge for the thermoregulatory and water systems of the player’s body, with reported sweating rates of 2.37 L/h and a possible loss of 4–8% of the body mass [17], the consumption of this kind of drinks is advisable during and after practicing sport in order to counteract the high sweating rates [16]. Likewise, isotonic drinks intake is especially advisable when finishing the effort, since liquids intake during the effort can only be completed during breaks between games, meaning that it is lower than the sweating rate, which leads to an increase of the instability among lost and ingested liquids [17].

Unlike badminton, a sport in which the consumption of milk whey protein is the highest, basically due to its impact on protein synthesis [18], the consumption of such protein is below 50% in squash players. It is worth mentioning that the recommended proteins intake in athletes is between 1.4 and 2 g/kg/day, and the proteins intake is advised to be completed during or after exercise, because of the positive effect observed on the increase of myofibrillar proteins synthesis and the decrease of myofibrillar damage and muscle pain sensation [19]. In fact, it has been suggested that proteins intake in peri-training implies a better recovering and higher increases of the lean mass [20]. This way, the intake of a protein supplement relating to exercise as part of a nutritional intervention has been proven to be effective in a case study of a female player aiming to reach a daily proteins intake of 1.4–1.6 g/kg/day and leading to improvements in her body composition and her physical performance [21].

Regarding ergogenic aids of group A, statistically significant differences were found (*p* = 0.035) between both groups concerning sodium bicarbonate, which was consumed by 28.6% of the international-level players and only by 3.6% of the national-level players. As for the rest of ergogenic aids of group A, though with no statistically significant differences, it was found that 28.6% of the international-level players and 39.3% of the national-level ones consume caffeine, 28.6% and 17.9% consume creatine and 14.3% of both of the groups consume β-alanine, whereas none is reported to consume nitrate salts or beetroot juice. These results suggest a non-appropriate NS consumption pattern, since the NS use rate with possible ergogenic effects does not exceed the 30%, with the exception of caffeine in international players, who show a consumption of 39.3%.

This way, supplementation with sodium bicarbonate improves blood pH regulation and it has been proven positive in efforts with a high glycolytic component, delaying muscle fatigue [22] or improving performance when facing sprints around 60 seconds long [8]. This way, it has been observed that in tennis such supplementation was effective in reducing fatigue and preventing performance from lowering in a simulated game, taking as evaluation parameters the serve precision, as well as combined forehand and backhand [22]. Regarding supplementation with β-alanine, it is effective for increasing the concentrations of muscular carnosine [23], a protein that acts at an intracellular level as a calcium transporter from the sarcoplasmic reticulum to the sarcoplasm, while capturing a H+ ion from the sarcoplasm and transporting it to the sarcolemma [24], resulting in a muscle contraction improvement due to an improvement of calcium bioavailability and a pH regulation at an intracellular level that prevents phosphofructokinase activity, and so glycolysis activity, from being inhibited [25]. Thus, supplementation with β-alanine has been effective for improving performance in high-intensity continuous exercises [8].

As for caffeine consumption in international-level squash players, it has been reported to be lower than in tennis players [15]. Caffeine acts as an important activator of the central nervous system, due to its effect as adenosine receptor antagonist, reducing effort perception during exercise and increasing the release of endorphins, and improving the neuromuscular function as well as that of the vigilance and alert state [8]. This way, it has been suggested that supplementation with caffeine has an ergogenic effect in racquet sports, such as tennis [26]. Supplementation with creatine, for its part, increases muscle creatine reserves, improving the rate of resynthesis of phosphocreatine during high-intensity efforts [8,9], while it increases cellular osmolarity, thus stabilising the cell membrane and reducing muscle damage [9]. Furthermore, it is the only NS which effect has been proven in squash players, having shown a positive effect on speed in high performance players, thereby being considered a NS with ergogenic effect in squash [1,8]. In this sense, supplementation with nitrate, in salts or beetroot juice, is effective for increasing the levels of nitric oxide in blood, which has, among its functions, a vasodilator effect, an increase of blood flow, and of mitochondrial efficiency and biogenesis, as well as being a muscle contractility enhancer. Supplementation with these compounds has been considered to be positive in intermittent dynamic efforts of high intensity, such as squash [27], having been proved to have ergogenic effects both on cardiorespiratory endurance categories [28] and facing high-intensity efforts [29].

Regarding the supplements of group B, statistically significant differences were detected among groups, where international-level players showed a higher intake of flaxseed oil (28.6% vs. 3.6%, *p* = 0.02), glutamine (50% vs. 17.9%, *p* = 0.03), and branched-chain amino acids supplements (57.1% vs. 21.4%, *p* = 0.02). According to the Australian Institute of Sport, this kind of supplements should be given to athletes under investigation or clinical monitoring, under supervision. However, higher consumption patterns have been observed in relation to the ones reported regarding the supplements of group A in squash players.

Relating to flaxseed oil, it should be pointed out that it is a food functional ingredient that us rich in α-linolenic acid (omega-3 fatty acid), lignans, and fibre to which health benefits, such as reducing the risk of cardiovascular disease, atherosclerosis, diabetes, cancer, arthritis, osteoporosis, and autoimmune and neurological disorders are attributed [30]. However, its impact on sports performance is still to be defined, although some point out a possible protection against bone mass loss [31] and the capacity of mobilising and using the fat as fuel during long training sessions [32].

Athletes are recommended to consume ≤3000 mg daily of polyunsaturated omega-3 fatty acids (EPA + DHA) that can be obtained from a varied diet that includes specially wholefoods, seeds, walnuts, sardines, and salmon, so that the supplementation with flaxseed oil would only be advisable for individuals with deficiencies in this sense or who are vegetarians [33].

Regarding glutamine consumption, it is significant that, although its effects on sports performance have not been confirmed and further research is required [8,34], it is the second most consumed ergogenic aid among squash players, way above group A supplements. It is a non-essential amino acid that acts as an energy substrate for cells in the immune system (mainly lymphocytes) and that it is reduced after prolonged exercise and tough training [34,35]. Although there is debatable evidence that supplementation with glutamine could have benefits on resistance exercise [34] or on delaying the appearance of delayed-onset muscle soreness [36], athletes usually consume it in order to strengthen their immune system, to increase the glycogen synthesis, or to achieve an anti-catabolic effect [35], but it has no impact on strength training [34] or on the immune function [8,35], thereby it would not be a recommended supplement in squash players.

In relation to branched-chain amino acids (isoleucine, leucine, and valine), it is significant to point out that it is the most consumed ergogenic aid by international-level squash players (57%), which is in line with what has been observed among Brazilian badminton athletes [18]. It seems that the consumption of such supplements is linked to the stimulation of protein synthesis, neural function improvement, and blood glucose and insulin adjustment [19]. Nevertheless, since the scientific evidence level regarding supplementation with branched-chain amino acids is lower, it is suggested that it could be more interesting to provide with a high biological value protein or with the supplementation of milk whey protein.

The purchase of NS can pose health risks due to: the lack of available information on these products [10,32]; the discovery of non-declared pharmacological substances on the labels [33]; inadequate use outside the optimal protocol [32]; or, to lacking current legislation [11]. Policies applied to sports nutrition recommend that legislation regarding NS should be specific thus allowing for knowledge of the benefits or limitations and evidence supporting their use in athletes, as many studies have revealed that many NS consumers are unaware of what they are taking and they may even be ingesting harmful NS or added damaging and/or banned products [11,35,36].

Regarding advice, 28.6% (4/14) of the international-level players state that the person responsible for advising them is their personal trainer and 21.4% (3/14) are advised by a dietitian-nutritionist, which shows statistically significant differences (*p* < 0.01) when compared to the national-level athletes, who follow mostly diets without any professional’s prescription (5/9, 55.6%). These results are in line with what has been pointed in the systematic revision on athletes’ nutritional knowledge, in which it is brought to light that athletes use a wide variety of nutritional information sources, including trainers and dietitians-nutritionists, but also books, specialised magazines, media, and Internet, or even elite competitions. We should consider that athletes who get advice from a dietitian-nutritionist as their main source of nutritional information have better dietary habits, as well as a better understanding of nutrients periodisation [37]. In addition, it has also been proven that athletes who go to dietitians-nutritionists show a higher consumption of NS that have demonstrated to have a high scientific level of evidence on their performance improvement effect [38].

The scientific literature reveals a lack of education for elite athletes in relation to the use of NS [39,40,41]. Accordingly, athletes need to be aware of the need for improved knowledge of the potential benefits and adverse effects of NS that is targeted at improving their health and nutrition state, as well as sports performance [42]. The consumption of a NS needs to be through the recommendation of a diet-nutrition specialist and based on the training, health and nutrition state of the athlete [43] as well as on the assessment of each NS within the legal framework [44].

## 5. Conclusions

International-level squash players show a higher NS consumption when compared to national-level players, which is in line with what has been proven in other sports categories in which a higher consumption among higher-level athletes has been observed [6], even though, unlike other racquet sports, such as tennis [11], 100% of the international-level squash players consume NS. It has been proven that, just like in other sports categories, only a minority of squash players are advised by dietitians-nutritionists [34]. In addition, the low consumption of NS with a potential ergogenic effect, such as creatine, sodium bicarbonate, caffeine, β-alanine, and nitrate/beetroot juice, which have been proven to have effects on sports performance [7,8], makes the NS consumption pattern to be imbalanced based on the level of scientific evidence. Since advice from dietitians-nutritionists makes the athletes’ NS consumption pattern more compliant with the requirements specific to each sports category [11], the fact of including dietitians-nutritionists in nutritional advice for squash players could have an impact on performance improvement.

## Figures and Tables

**Table 1:** Descriptive values of international-level and national-level players.

Variable	International	National	*T*	*p*
Age (years) *	25.0 ± 6.2	35.6 ± 14.2	−2.66	0.01
Weight (kg)	72.1 ± 10.1	68.3 ± 11.4	1.06	0.29
Size (m)	1.8 ± 0.1	1.7 ± 0.1	1.60	0.13
BMI (kg/m^2^)	22.5 ± 1.3	22.4 ± 2.6	0.06	0.95
Weekly training sessions *	5.6 ± 1.2	3.9 ± 1.6	3.54	<0.01

BMI = body mass index; Data shown as Mean (M) + Standard Deviation (SD) *; Statistically significant differences among groups (*p* < 0.05).

**Table 2:** Consumption of nutritional supplements by international-level and national-level athletes in the different categories established by Australian Institute of Sport (AIS).

Category	Supplement	International		National		*p* Value
		*n*	%	*n*	%	
Sports food	Energy bars	11	78.6	14	50.0	0.08
	Isotonic drinks	10	71.4	14	50.0	0.19
	Carbohydrates gainers	1	7.1	2	7.1	1.00
	Electrolytes	5	35.7	4	14.3	0.11
	Malto-dextrines	0	0	2	7.1	0.31
	Meat protein	2	14.3	2	7.1	0.46
	Milk whey protein	6	42.9	8	28.6	0.36
	Casein	0	0	1	3.6	0.47
	Vegetable protein	1	7.1	2	7.1	1.00
Medical supplements	Mineral complex	3	21.4	1	3.6	0.06
	Vitamin complex	7	50.0	7	25.0	0.11
	Iron	5	35.7	4	14.3	0.11
	Probiotics	1	7.1	1	3.6	0.63
	Vitamin D	4	28.6	3	10.7	0.14
Ergogenic aids of group A	B-alanine	2	14.3	2	7.1	0.46
	Bicarbonate of soda *	4	28.6	1	3.6	0.04
	Caffeine	4	28.6	11	39.3	0.50
	Creatine monohydrate	4	28.6	5	17.9	0.43
	Creatine alkaline	0	0	1	3.6	0.47
Ergogenic aids of group B	ω-3 fatty acids	1	7.1	4	14.3	0.50
	Cod liver oil	1	7.1	1	3.6	0.61
	Flaxseed oil *	4	28.6	1	3.6	0.02
	Evening primrose oil	1	7.1	1	3.6	0.61
	Acetyl-L-carnitine	2	14.3	1	3.6	0.20
	L-carnitine	0	0	5	17.9	0.09
	Shark cartilage	0	0	4	14.3	0.14
	Chondroitin	0	0	2	7.1	0.31
	Curcumin	0	0	1	3.6	0.47
	Glucosamine	1	7.1	2	7.1	1.00
	Glutamine *	7	50.0	5	17.9	0.03
	Branched-chain amino acids *	8	57.1	6	21.4	0.02
	Vitamin C	5	35.7	7	25.0	0.47
	Vitamin E	4	28.6	3	10.7	0.14
Ergogenic aids of group C	ω-6 fatty acids	0	0	3	10.7	0.20
	ω-9 fatty acids	0	0	1	3.6	0.47
	Coconut oil	3	21.4	2	7.1	0.18
	Essential amino acids	2	14.3	4	14.3	1.00
	Arginine	0	0	3	10.7	0.20
	Chitosan	2	14.3	1	3.6	0.22
	Zinc	0	0	3	10.7	0.20
	Citrulline malate	0	0	1	3.6	0.47
	Spirulina	1	7.1	1	3.6	0.61
	Pre-workout formulas	2	14.3	2	7.1	0.46
	Ginseng	3	21.4	4	14.3	0.56
	Guarana	3	21.4	2	7.1	0.18
	Royal jelly	3	21.4	7	25.0	0.80
	Soy lecithin	1	7.1	0	0	0.15
	Leucine	1	7.1	1	3.6	0.61
	Beer yeast	2	14.3	1	3.6	0.20
	Magnesium	6	42.9	6	21.4	0.15
	Melatonin	0	0	1	3.6	0.47
	Pollen	2	14.3	2	7.1	0.46
	Taurine	1	7.1	4	14.3	0.50
	Green tea	2	14.3	7	25.0	0.43
	Vitamin K	2	14.3	1	3.6	0.20
	Collagen	0	0	1	3.6	0.47

* Statistically significant differences among groups (*p* < 0.05).
